# Screening and diagnosis of metabolic dysfunction-associated steatotic liver disease in children within public healthcare

**DOI:** 10.1038/s41390-025-04540-w

**Published:** 2025-10-30

**Authors:** Lyydia Lahtinen, Pauliina Hiltunen, Nina Vuorela, Heini Huhtala, Kalle Kurppa, Linnea Aitokari

**Affiliations:** 1 Tampere Center for Child, Adolescent and Maternal Health Research, Faculty of Medicine and Health Technology, Tampere University, Tampere, Finland; 2 Celiac Disease Research Center, Faculty of Medicine and Health Technology, Tampere University, Tampere, Finland; 3 Department of Pediatrics, Tampere University Hospital, Wellbeing Services County of Pirkanmaa, Tampere, Finland; 4 Faculty of Social Sciences, Tampere University, Tampere, Finland; 5 The University Consortium of Seinäjoki, Seinäjoki, Finland; 6 Valkeakoski Social and Healthcare Center, Wellbeing Services County of Pirkanmaa, Valkeakoski, Finland

## Abstract

**Background:**

Metabolic dysfunction-associated steatotic liver disease (MASLD) has increased rapidly. NASPGHAN recommends screening children at risk for MASLD, but its implementation remains unclear. We explored the implementation of screening in healthcare.

**Methods:**

Data were collected from 908 children (median 11.4 years, 42.7% girls) investigated for overweight/obesity. Emphasis was placed on screening methods, diagnosis and potential secular changes. The diagnosis was confirmed retrospectively using novel criteria.

**Results:**

Investigations included measuring alanine aminotransferase (ALT) in 83.1%, ultrasonography in 9.1%, and no screening in 16.5%. Screening was conducted in 86.2% of children meeting the recommendations, but 69.3% of those not eligible, mostly due to young age, were also screened. Screening was more frequent in tertiary care. Overall, 87.9% were tested for metabolic comorbidities and 80.9% underwent differential diagnostics, with 5.7% having other liver-affecting diseases/medications. ALT testing increased in tertiary care from 81.9% in 2002-2011 to 91.4% in 2012-2020 (*p* < 0.001), and exclusion of liver-affecting conditions improved in both tertiary (79.6% vs. 87.4%, *p* = 0.017) and primary care (61.5% vs. 84.6%, p < 0.001). MASLD was diagnosed clinically in 7.4%, while 14.5% met the criteria retrospectively.

**Conclusions:**

MASLD screening practices are mostly aligned with the recommendations. However, many patients remain unrecognized, underscoring the need for better guidelines.

**Impact:**

- In a large cohort of 3-17-year-old children investigated in healthcare due to obesity, implementation of MASLD screening largely aligned with current recommendations supporting general acceptance of screening.
- Screening was conducted in 86.2% of children meeting the NASPGHAN screening recommendations, while 87.9% were also tested for metabolic comorbidities, and 80.9% underwent differential diagnostics.
- MASLD was diagnosed clinically in 7.4%, while 14.5% met the criteria retrospectively, suggesting possible underdiagnosis.
- Compared to NASPGHAN guidelines, screening appeared excessive among younger children. Nevertheless, MASLD was also prevalent in this age group, indicating a need for further research to determine optimal age thresholds.

## Introduction

Pediatric overweight and obesity have become major global health issues affecting hundreds of millions of children and adolescents worldwide^1,2^. Consequently the prevalence of obesity-related metabolic dysfunction-associated steatotic liver disease (MASLD), previously known as non-alcoholic fatty liver disease (NAFLD), has also increased dramatically^3–6^. Although the risk varies across populations, MASLD as a whole may affect up to one-third of children with obesity^7,8^. They are concurrently susceptible to various other cardiometabolic complications^9,10^. Pediatric MASLD can be particularly aggressive and rapidly progressing^11,12^, further highlighting the importance of early diagnosis.

Complicating case-finding, MASLD may remain asymptomatic for a long time^13^. Therefore many guidelines, including those by the European (ESPGHAN) and North American (NASPGHAN) Societies for Pediatric Gastroenterology, Hepatology, and Nutrition and the Finnish Current Care Guidelines recommend active screening for the condition among at-risk children and adolescents^14–16^. Primary healthcare providers should play a central role in first-line screening for MASLD, while patients suspected of having more advanced forms should be referred to tertiary care^15–17^. Earlier studies indicate that the implementation of these recommendations varies widely, with MASLD still significantly underdiagnosed^18–20^, but the data remain limited.

In Finland, 26% of boys and 17% of girls aged 2–16 were overweight, and 8% and 3% were obese in 2023^21^. Associated MASLD appears to be common^8^. The availability of free public healthcare, routine health screenings for children and systematically maintained patient records in Finland yield invaluable data for research purposes. We exploited these factors to investigate the current screening and diagnostic practices for pediatric MASLD in public healthcare.

## Methods

### Patients and study design

The study was conducted at the Tampere Center for Child, Adolescent, and Maternal Health Research, Tampere University, at Tampere University Hospital (TaUH), and at the Primary Healthcare Unit in the City of Tampere. The study cohort comprised consecutive children aged 3–17 years of age who had received obesity-related ICD-10 diagnostic codes (E65, E66.0-9, and R63.5) at the Primary Care Unit in 2006–2020 or at TaUH in 2002–2020. Their systematically documented medical data at the time of first obesity-related investigations were gathered from the patient records. Possible laboratory investigations, imaging, and other relevant studies conducted within one year of the first associated healthcare visit were also collected. Degree of obesity was verified retrospectively as described below, and individuals proving to be of normal weight were excluded, likewise those with lacking anthropometric data (Fig. S1).

The study design and collection of patients’ register data were approved by the City of Tampere Healthcare Services, TaUH, and the Health Data Permit Authority Findata (permissions THL/246/14.02.00/2021 and THL/2656/14.06.00/2024). According to the national ethical and data processing guidelines, the study required no separate ethics committee approval as there was no intervention, and no subjects were directly contacted. The Declaration of Helsinki principles were strictly followed. Due to strict national data protection regulations, exact results for fewer than five individuals are not disclosed.

### Patient data

The following clinical information was collected at the time of the first relevant healthcare visit: demographic and anthropometric data, blood pressure and presence of acanthosis nigricans, prediabetes, lipid metabolic disorders and liver-affecting diseases or medications as recorded by the clinician. Hypertension was defined as systolic or diastolic blood pressure greater than the 95th percentile for age, sex and height^22^. Possible use of supplements, herbal products, alcohol, or illicit drugs and diagnoses of NAFLD or MASLD and type 2 diabetes (T2D) in first-degree relatives were also recorded.

Anthropometric data included weight, height, body mass index (BMI) Z-score and weight-to-height percentage. Overweight, obesity, or severe obesity were classified based on BMI Z-scores as recommended by the International Obesity Task Force^23^. Additionally, weight-to-height percentages (WH%) were utilized for children with WH% available, but missing exact weight or height data prevented the calculation of BMI Z-score (*n* = 73)^24^. Cutoff values for BMI Z-scores were >1.16 for overweight, >2.11 for obesity, and >2.76 for severe obesity in girls, and >0.78, >1.70, and >2.36 in boys based on national data^24^. These values correspond to BMI thresholds of >25 kg/m^2^, >30 kg/m^2^, and >35 kg/m^2^ for individuals aged ≥18 years^23,24^. Equivalent values for WH% were 10–20% for overweight, 20–40% for obesity, and >40% for severe obesity in those <7 years old, and respectively 20–40%, 40–60%, and >60% for older participants^24^.

The following laboratory values were collected: alanine aminotransferase (ALT), fasting glucose (reference value < 5.6 mmol/l), 2-hour values of oral glucose tolerance test (OGTT, <7.8 mmol/l), glycated hemoglobin (HbA1c, <39 mmol/l), high-density lipoprotein (HDL, ≥1.04 mmol/l) and low-density lipoprotein (LDL, <3.36 mmol/l) cholesterol and triglycerides (< 1.13 before and <1.47 mmol/l after the age of 10 years)^25–28^. The results of possible relevant imaging studies, including ultrasonography, magnetic resonance imaging (MRI), transient elastography, and computed tomography, as well as liver biopsy, were also obtained.

Laboratory exclusion of other liver-affecting diseases included testing for one or more of the following: biliary diseases (γ-glutamyl transferase, alkaline phosphatase, unconjugated and conjugated bilirubin), gastrointestinal diseases (serum immunoglobulin A, tissue transglutaminase 2 antibodies, endomysial antibodies, fecal calprotectin), hepatotropic viruses, inflammatory markers (C-reactive protein, erythrocyte sedimentation rate, white blood cell count), kidney diseases (creatinine, urea), liver function tests (international normalized ratio, prothrombin time, activated partial thromboplastin time, albumin, prealbumin), muscular diseases (creatine kinase), and thyroid diseases (thyroid-stimulating hormone, free thyroxine).

### Definitions for NAFLD and MASLD

Possible clinical diagnosis of NAFLD/MASLD was recorded. The diagnosis was based on liver biopsy, biochemical markers, and/or imaging, along with the exclusion of other liver-affecting conditions^14,15^. The updated criteria for MASLD were published in 2023, and were thus applied retrospectively^5,6^. They included ALT >2x the upper limit of normal (ULN; reference <25 U/L for boys and <22 U/L for girls) or steatosis detected histologically or via imaging, along with the presence of either overweight or obesity^5,6^. Diagnosis of steatohepatitis and fibrosis was based on liver biopsy. ALT levels exceeding 80 U/L were collected separately due to the previously reported increased risk of advanced MASLD^14,15^.

### Risk factors for MASLD

Prevalence of additional risk factors beyond obesity, as mentioned in the ESPGHAN and NASPGHAN recommendations, was noted, acknowledging that some of the children in the study had been investigated before these were published. At-risk individuals, according to ESPGHAN, included those with increased waist circumference, sleep apnea, presence of acanthosis nigricans, or family history of MASLD, obesity or T2D^14^. NASPGHAN identifies at-risk individuals as those with increased waist circumference, insulin resistance, pre-diabetes or diabetes, dyslipidemia, sleep apnea, and a family history of NAFLD^15^. All risk factors were defined in accordance with the guidelines. Both recommendations also identified ethnicity, particularly Hispanic origin, as a risk factor^14,15^. However, this was excluded from the analyses due to a lack of systematic ethnicity data.

According to the NASPGHAN guidelines, all children with obesity and those with overweight and additional risk factors should be screened for MASLD starting at the age of 9–11 years^14^. Additionally, screening may be conducted on younger children with severe obesity or family history of NAFLD^14^. We compared the recommendation to the implementation of screening practices, even though this was published after some children had already been screened. Only NASPGHAN guidelines were used for this purpose; ESPGHAN provides no unequivocal recommendations for screening^14^. The Finnish Current Care Guidelines for obesity, first published in 2005, recommend ALT testing in children with obesity but include no precise screening criteria^16^.

### Statistics

Distributions of continuous variables were assessed for normality by visual examination and, when appropriate, by Shapiro-Wilk and Kolmogorov-Smirnov tests. Variables with Gaussian distribution were presented as mean, standard deviation, and range, and skewed variables as median, quartiles, and range. Prevalences of children undergoing examinations or presenting with NAFLD/MASLD were reported as numbers and percentages. Group comparisons were conducted using the Mann-Whitney test for non-Gaussian continuous variables, the t-test for Gaussian continuous variables, and the Chi-square test for categorical variables. When the assumptions for Chi-square test were not met, Fisher’s exact test was used. Changes over time in screening and diagnoses were analyzed by Chi-square test and limited to the years 2006-2020 due to small numbers of participants in earlier years and lack of primary care data. Statistical significance was defined as P value < 0.05. All analyses were performed using SPSS version 29.0 (Armonk, NY: IBM Corp).

## Results

After excluding six children with missing data, such as anthropometric measurements, and five children re-classified as being of normal weight, the final cohort comprised 343 patients from primary care and 565 from tertiary care (Table 1). The children in the study had ALT levels ranging from low normal to >200 U/L. Altogether, 334 were aged 3–10, and 574 were aged >10. Children investigated in tertiary care were older, had higher ALT levels, presented more often with prediabetes, dyslipidemia, acanthosis nigricans, and ESPGHAN/NASPGHAN-defined risk factors, and were more likely to have a first-degree relative with T2D (Table 1). NASPGHAN screening criteria were met by 751 children, representing 82.7% of the total cohort.

**Table 1:** Characteristics of 908 children with overweight or obesity, further categorized by the healthcare site responsible for medical examinations

	All children n = 908		Tertiary care n = 565		Primary care n = 343	
	n	%	n	%	n	%
Age, median (quartiles), yr	11.4	8.5, 13.7	11.9	9.2, 14.2^**^	10.4	7.7, 12.4
BMI Z-score, mean (SD)	2.5	(0.6)	2.5	(0.6)	2.5	(0.6)
ALT, median (quartiles), U/L	25.0	19.0, 37.0	26.0	19.0, 40.0^**^	23.0	18.0, 31.0
Girls	388	42.7	234	41.4	154	44.9
Severe obesity^a^	415	45.7	267	47.3	148	43.1
Hypertension^b^	349	38.4	222	39.3	127	37.0
Prediabetes^c^	280	30.8	191	33.8^*^	89	25.9
Dyslipidemia^d^	368	40.5	262	46.4^**^	106	30.9
Acanthosis nigricans	82	9.0	77	13.6^**^	5	1.5
Sleep apnea	30	3.3	30	5.3^**^	0	0
Liver diseases in FDR	6	0.7	6	1.1	0	0
T2D in FDR	86	9.5	70	12.4^*^	16	4.7
ESPGHAN risk factors^e^	462	50.9	335	59.3^**^	127	37.0
NASPGHAN risk factors^f^	561	61.8	374	66.2^**^	187	54.5

^a^As defined by Cole et al.^23^ and Saari et al.^24^; ^b^blood pressure >95th percentile^22^; ^c^as defined by American Diabetes Association^25^; ^d^increased level of any of the following: triglycerides, total cholesterol and high-density lipoprotein or low-density lipoprotein cholesterol^26^; ^e^increased waist circumference, acanthosis nigricans or family history of NAFLD, obesity or T2D; ^f^the ESPGHAN criteria, as well as presence of hypertension, prediabetes or dyslipidemia. Only measurements/imaging conducted within one year after the first obesity-related visit are considered. ALT, alanine aminotransferase; BMI, body mass index; FDR, first degree-relative; T2D, type 2 diabetes; ESPGHAN, The European Society for Pediatric Gastroenterology Hepatology and Nutrition; NASPGHAN, the North American Society for Pediatric Gastroenterology Hepatology and Nutrition; NAFLD, non-alcoholic fatty liver disease. Data on BMI Z-score, ALT, hypertension, prediabetes, dyslipidemia, acanthosis nigricans and sleep apnea were available for 78–83% of the children in the study. Statistical significance between specialized care and primary care; *P < 0.05, **P < 0.001

### Investigations conducted in healthcare and secular trends

**Fig. 1:**
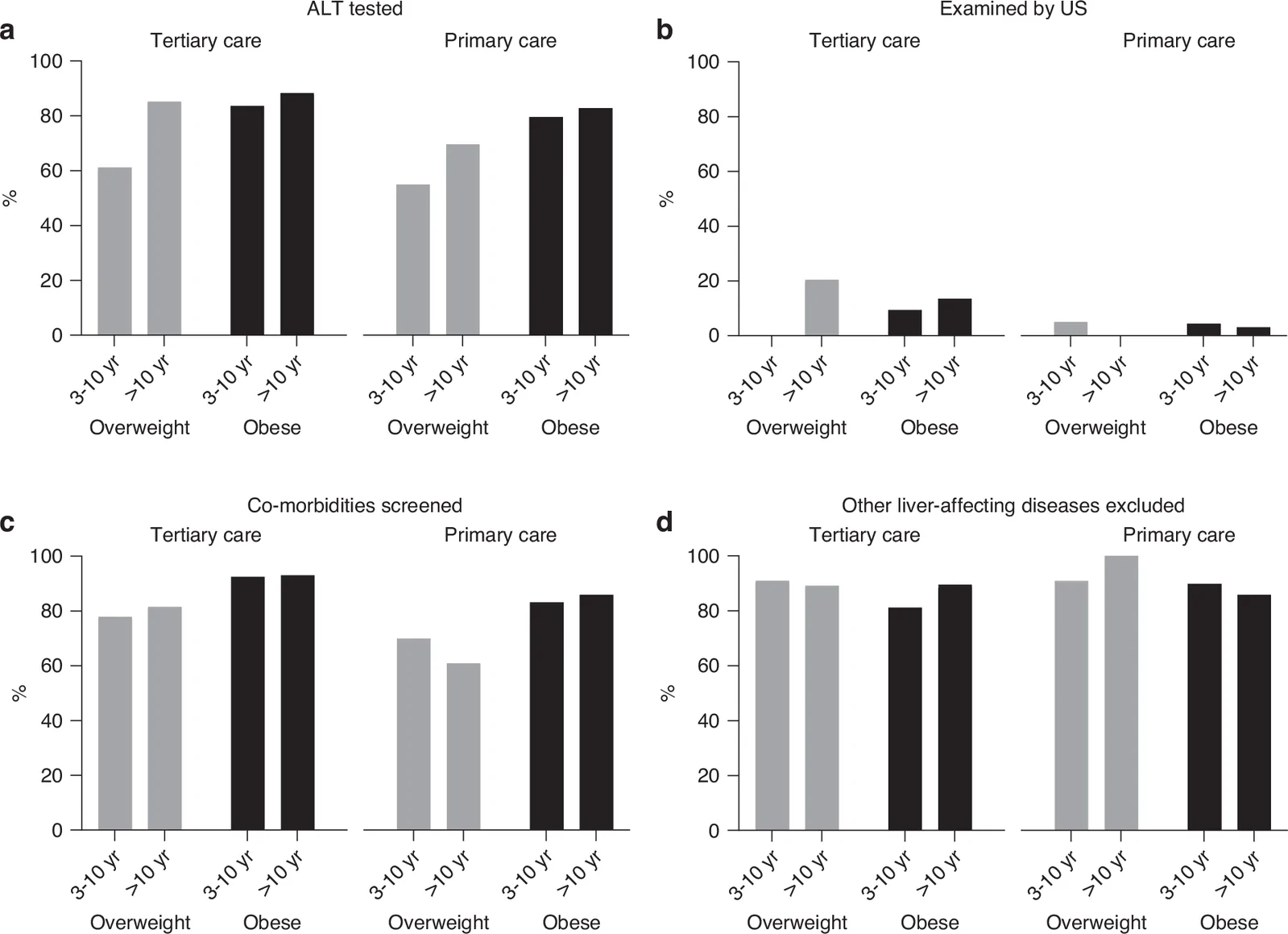
Investigations of 908 children with overweight or obesity, further categorized by age and healthcare site. Screening for co-morbidities included measurement of one or more of the following: fasting glucose, oral glucose tolerance test, glycated hemoglobin, fasting insulin, total cholesterol, high-density lipoprotein or low-density lipoprotein cholesterol, and triglycerides. Other liver-affecting diseases were deemed excluded if mentioned in the medical records or if any of the following laboratory measurements had been conducted: liver function tests, celiac autoantibodies, fecal calprotectin, thyroidal testing, markers of hepatotropic viruses, creatine kinase, blood lactate, C-reactive protein, creatinine, and urea. Only measurements/imaging conducted within one year after the first obesity-related visit were considered. ALT alanine aminotransferase, US ultrasonography.

Screening was more frequent in older children and in those in tertiary care (Fig. 1). ALT was tested in 83.1% (79.0% in those aged 3-10 and 85.5% in those >10 years; p = 0.012) and 9.1% underwent ultrasonography (6.6% vs. 10.6% respectively; p = 0.042). Altogether 8.8% underwent both ALT testing and ultrasonography, with ALT testing conducted first in 62.5% of cases. Screening for liver steatosis was not performed in 16.5% of cases (20.7% vs. 14.1% respectively; *p* = 0.010). MASLD screening was conducted in 86.2% of children meeting the NASPGHAN screening criteria; however, 70 (69.3%) of those who did not meet the criteria were also screened. Among those not meeting the criteria, 70.7% were 3–10 years old, and 65.0% were girls. Fewer than five children did not participate in the recommended laboratory testing in tertiary care, while in primary care there were no such cases.

Other obesity-related comorbidities were screened in 87.9% (86.5% vs. 88.7% respectively; *p* = 0.339), of whom 9.5% were not screened for MASLD. Any exclusions for other liver-affecting conditions were conducted in 80.9% (78.4% vs. 82.4% respectively; *p* = 0.143). These included thyroid diseases in 86.5%, inflammatory markers in 81.0%, kidney diseases in 45.9%, biliary diseases in 20.0%, gastrointestinal autoimmune disease in 10.9%, liver function tests in 7.2%, creatine kinase in 1.6%, and exclusion of hepatotropic viruses in 1.0%.

**Fig. 2:**
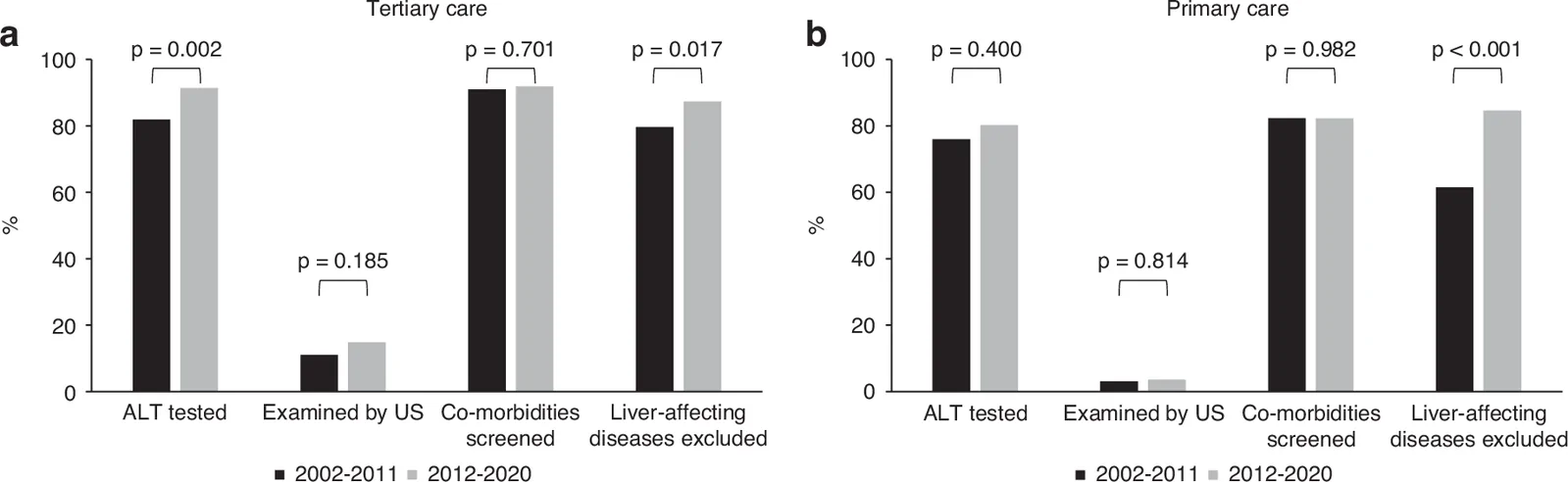
Screening and diagnostic methods of metabolic dysfunction-associated steatotic liver disease (MASLD) among 908 children with overweight or obesity in 2002–2011 and 2012–2020, further categorized by the healthcare site of their first visit. Screening for co-morbidities included any of the following measurements: fasting glucose, fasting insulin, glycated hemoglobin, oral glucose tolerance test, total cholesterol, high-density lipoprotein or low-density lipoprotein cholesterol and triglycerides. Other liver-affecting diseases were deemed excluded if mentioned in the medical records or any of the following laboratory measurements were conducted: liver function tests, celiac autoantibodies, fecal calprotectin, thyroid-stimulating hormone, free thyroxine, markers of hepatotropic viruses, creatine kinase, blood lactate, C-reactive protein, creatinine, and urea. Only measurements/imaging conducted within one year after the first obesity-related visit were considered. ALT alanine aminotransferase, US ultrasonography.

ALT testing increased significantly in tertiary care, from 81.9% in 2002–2011 to 91.4% in 2012–2020, and exclusion of liver-affecting conditions increased in both tertiary (79.6% to 87.4%) and primary care (61.5% to 84.6%) (Fig. 2).

### Abnormal screening results, children receiving diagnoses, and secular trends in diagnoses

ALT >2x ULN was seen in 16.7% of those tested (8.3% in those 3–10 and 21.1% in those >10; *p *< 0.001). ALT > 80 U/l was observed in 5.2%. Liver steatosis was detected via ultrasonography in 65.1% of participants, (50.0% vs. 70.5%, respectively; *p* = 0.084). Among those with ALT <2x ULN, 31.4% had steatosis in ultrasonography, while 12.5% with ALT <1x ULN also showed steatosis. In contrast, 4.4% of those with ALT >2x ULN and 18.8% of those with ALT >1x ULN had no steatosis in ultrasonography. Fewer than ten children were examined with MRI or underwent liver biopsy, all having ALT >2x ULN. Steatosis was observed in under five MRIs, and steatohepatitis with F1-2 fibrosis in under five biopsies, all of which were associated with ALT levels >80 U/L. The indication for biopsy was either suspicion of more advanced MASLD or other liver-affecting condition. The mean age of patients undergoing biopsy was 11.5 years; all were classified as obese, and the majority were severely obese. ALT levels were measured either prior to or on the day of the biopsy, and most patients underwent ultrasonography before the procedure. None of the children were examined with computed tomography or transient elastography.

Overall, 7.4% of children received a NAFLD/MASLD diagnosis from a clinician (3.9% of those aged 3–10 and 9.4% of those >10 years; *p* = 0.012), with clinicians using biochemical markers, imaging, or biopsy as diagnostic methods after excluding the possibility of other diseases. However, 14.5% met the MASLD criteria when assessed retrospectively using similar criteria (7.8% vs. 18.5% respectively; *p* < 0.001). Among children meeting the MASLD screening criteria according to NASPGHAN, 8.3% were diagnosed with NAFLD and 15.8% met the MASLD criteria. In contrast, among those who did not meet the screening criteria, 1.0% were diagnosed with NAFLD and 4.0% met the MASLD criteria.

Other liver-affecting diseases were found in 3.2% of the patients, 31.0% of them also fulfilling the MASLD criteria, while 2.5% were taking liver-affecting medications or substances, 26.1% of them with MASLD. Other liver-affecting diseases or medications were comparable between age groups (6.9% in those 3–10 and 5.1% in those >10; *p* = 0.251). Other liver-affecting diseases were alpha-1 antitrypsin deficiency, autoimmune thyroid disease, celiac disease, ulcerative colitis, Duchenne muscular dystrophy, Down syndrome, Klippel–Trénaunay–Weber syndrome, mitochondriopathy, panhypopituitarism, Prader–Willi syndrome and Turner syndrome. Liver-affecting medications or substances included alcohol abuse, antiepileptic medications, antipsychotic medications, and rheumatologic medications.

**Fig. 3:**
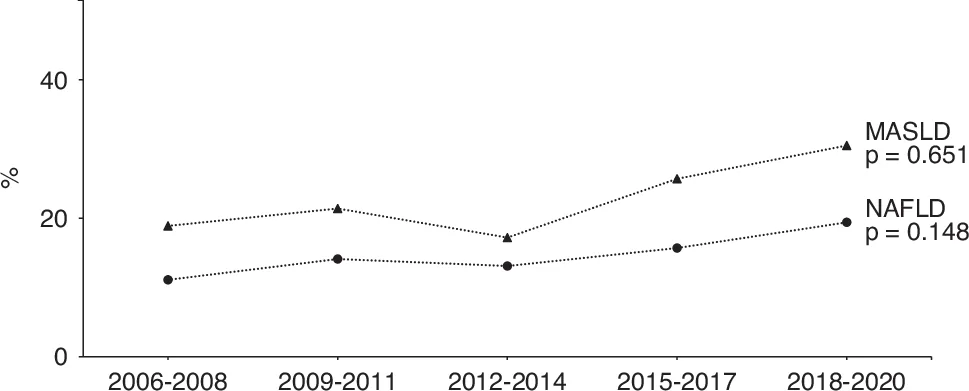
Secular trends in the prevalence of clinically diagnosed NAFLD and retrospectively identified cases meeting the criteria for MASLD among 908 children with overweight or obesity who had their first obesity-related healthcare visit in different years. The diagnosis were based on liver biopsy, biochemical markers, and/or imaging, along with the exclusion of other liver-affecting conditions. NAFLD non-alcoholic fatty liver disease, MASLD metabolic dysfunction-associated steatotic liver disease.

The prevalence of children with NAFLD diagnosed by clinicians increased slightly over time, also in those who retrospectively met the MASLD criteria (Fig. 3).

## Discussion

Overall, 83.1% of children underwent ALT testing, and 9.1% were assessed with ultrasonography. Although the recommendations were published only towards the end of the study period, 86.2% of children meeting the NASPGHAN recommendations were screened for MASLD. The ESPGHAN criteria were published in 2012, and accordingly, we found that ALT testing in tertiary care had increased thereafter. Before this, our Finnish Current Care Guidelines, available since 2005, mentioned ALT testing as part of the investigation of obese children, but included no explicit screening indications, which may have caused confusion among clinicians^16^. No increase in ultrasonography use although this is recommended by ESPGHAN^14^. Comparable studies are scarce, but, like us, Draijer et al. reported that 83.5% of Dutch children aged 7-12 attending an obesity clinic underwent ALT screening^29^. In contrast, Gulati et al.^18^ found that only 9.3% of children in the USA aged 10-14 eligible for the screening were tested, while in the study by Morkem et al.^19^, this was performed in 23.6% of 9–18-year-old Canadian children. Research on investigations other than ALT testing is limited, but in a European survey 79.1% of pediatricians reported using ultrasonography, 9.5% MRI, and 13.9% liver biopsy^30^.

As stated by the World Health Organization, any screening must be acceptable to the population^31^. In our study, overall acceptance of MASLD screening was excellent and generally higher than previously reported^18,19^. In fact, only a few families did not attend the recommended investigations. Draijer et al. (2022) reported similarly high acceptance rates in a specialized obesity clinic, but this may not reflect the situation in primary healthcare^29^. The substantial variation in MASLD screening rates may be attributed, at least in part, to differences in healthcare structure and access, ethnicity, and other factors^18^.

Altogether, 16.7% of the children had ALT >2x ULN, and 65.1% of those who underwent ultrasonography were diagnosed with hepatic steatosis. These results suggest that screening is generally effective in detecting abnormalities. Nevertheless, combining ultrasonography with ALT testing did not improve detection rates when using a cutoff >1x ULN, which is consistent with the limited sensitivity of ultrasonography for detecting mild steatosis^15,32^. Nevertheless, in line with Ezaizi et al.^33^, the combination of ultrasonography and ALT identified additional MASLD cases when the threshold was raised to >2x ULN. Due to poorly defined ALT cutoffs and the variable sensitivity of ultrasonography^32,33^, ESPGHAN recommends combined investigations, whereas NASPGHAN and the Finnish Current Care Guidelines advocate ALT testing alone^14–16^.

Notably, 69.3% of children who did not meet the NASPGHAN criteria, particularly younger children, still underwent screening for MASLD. This may be partly due to the lack of a defined screening age in the ESPGHAN recommendations and in the Finnish Current Care Guidelines^14,16^. In contrast to some earlier studies^34^, ALT >2x ULN was also frequent in these children, and a significant number of NAFLD/MASLD cases were identified among them. This may suggest that even younger children should be screened, but the data on the prevalence of MASLD in this age group are limited. Further research is needed to determine the optimal practices and appropriate age thresholds for efficient MALSD screening that would not unnecessarily burden healthcare resources.

Overall, 87.9% of the children were screened for glucose and lipid metabolism disorders, in accordance with the ESPGHAN and NASPGHAN guidelines, which emphasize the importance of investigating these associated conditions in individuals suspected of having MASLD^14,15^. The Finnish Current Care Guidelines also recommend screening for these disorders in all children with obesity^16^. Conversely, more active MASLD screening in children with metabolic abnormalities has also been observed^20,35^. At present, despite their frequent coexistence^8^, the screening guidelines for MASLD, prediabetes, and dyslipidemia are all separate^14,15,25^, which is somewhat impractical and confusing. A unified strategy could streamline the process and improve cost-effectiveness.

The majority of children underwent at least some exclusion tests for hepatic co-morbidities, although this process was quite unsystematic. This variability was also evident in children aged under 10 years, for whom ESPGHAN specifically recommends differential diagnostics^14^. On a positive note, exclusion rates improved over time. Younossi et al. found that 65% of specialists and 55% of primary care physicians attempted to exclude the possibility of other liver diseases in children with suspected NAFLD^35^. In our study, the prevalence of coexisting conditions was consistent across age groups, thereby underscoring the need for a systematic exclusion strategy for all. Furthermore, an established liver condition could cause coexisting MASLD to be missed, particularly in tertiary care settings. Recent reviews have proposed comprehensive guidelines for differential diagnosis, but improving clinicians’ awareness is also necessary^36^.

The reported prevalence of MASLD varies widely, likely due to differences in screening methods, diagnostic criteria, and study populations^4,7,8^. Here, 7.4% of the children had received a NAFLD diagnosis from clinicians, while 14.5% met the MASLD criteria retrospectively. Notably, 11.4% of these individuals had liver-related diseases or potentially hepatotoxic medications or substances, which were exclusion criteria for NAFLD^14,15^, while MASLD can by definition co-occur with these conditions^5,6^. This complicates the interpretation of the findings, but significant underdiagnosis is apparent, as has been previously noted^37,38^. Clinicians used various diagnostic approaches, including biochemical markers, imaging, or biopsy as diagnostic methods after excluding the possibility of other diseases, likely reflecting both the heterogeneity of diagnostic recommendations and the limited availability of imaging in primary care. Of note, liver biopsy was performed in only a few cases, which is understandable given the invasive nature of the procedure. The finding that 5.2% of patients had ALT > 80 U/l suggests under-recognition of advanced forms of MASLD^14,15^. None of the patients underwent transient elastography, as this technique has only recently become available at the study site. Nevertheless, ultrasound-based elastography techniques offers a promising approach to improve MASLD screening and non-invasive fibrosis detection in the future^39,40^.

Our strengths were a large, well-defined study cohort and the availability of comprehensive medical data. The inclusion of patients from primary healthcare settings broadened the scope of the study and improved its generalizability. The main limitation was the retrospective design. Furthermore, the cohort was selected based on overweight-related diagnostic codes, potentially introducing selection bias^38^. Additionally, changes in screening guidelines and clinical practices over time may have increased heterogeneity. The recent availability of transient elastography has changed MASLD diagnostics, and its absence in this study may restrict the generalizability of our findings. The design used also prevented us from obtaining detailed information on clinical decision-making regarding the diagnosis of NAFLD and the exclusion of other conditions. Finally, the ethnically homogenous population of Finland and the availability of free public healthcare for children should be considered when interpreting the results.

## Conclusions

The implementation of MASLD screening in Finnish children largely aligned with the current recommendations. However, many affected children may go unrecognized, partially due to asymptomatic presentation. Screening also appeared excessive, particularly among younger children, in light of the current guidelines. Nevertheless, MASLD was also prevalent in this age group, indicating a need for further research to determine optimal age thresholds. Practical, evidence-based screening guidelines, a potential unified screening strategy for MASLD and other obesity-related metabolic disorders, along with improved clinician awareness are essential for the early identification and intervention to prevent advanced forms of MASLD.

## Supplementary information


Supplementary Information


## Data Availability

The data that support the findings of this study are available from Findata but restrictions apply to the availability of these data, which were used under license for the present study, and so are not publicly available. Data are, however, available from the authors upon reasonable request and with permission from Findata.
