# Supplementary material for: Screening and diagnosis of metabolic dysfunction-associated steatotic liver disease in children within public healthcare
Source: Pediatr Res. 2025 Oct 30;100(1):263–9. doi: 10.1038/s41390-025-04540-w (PMC13524811; doi:10.1038/s41390-025-04540-w)
Supplement: Supplementary file 1 [file 41390_2025_4540_MOESM1_ESM.pdf]

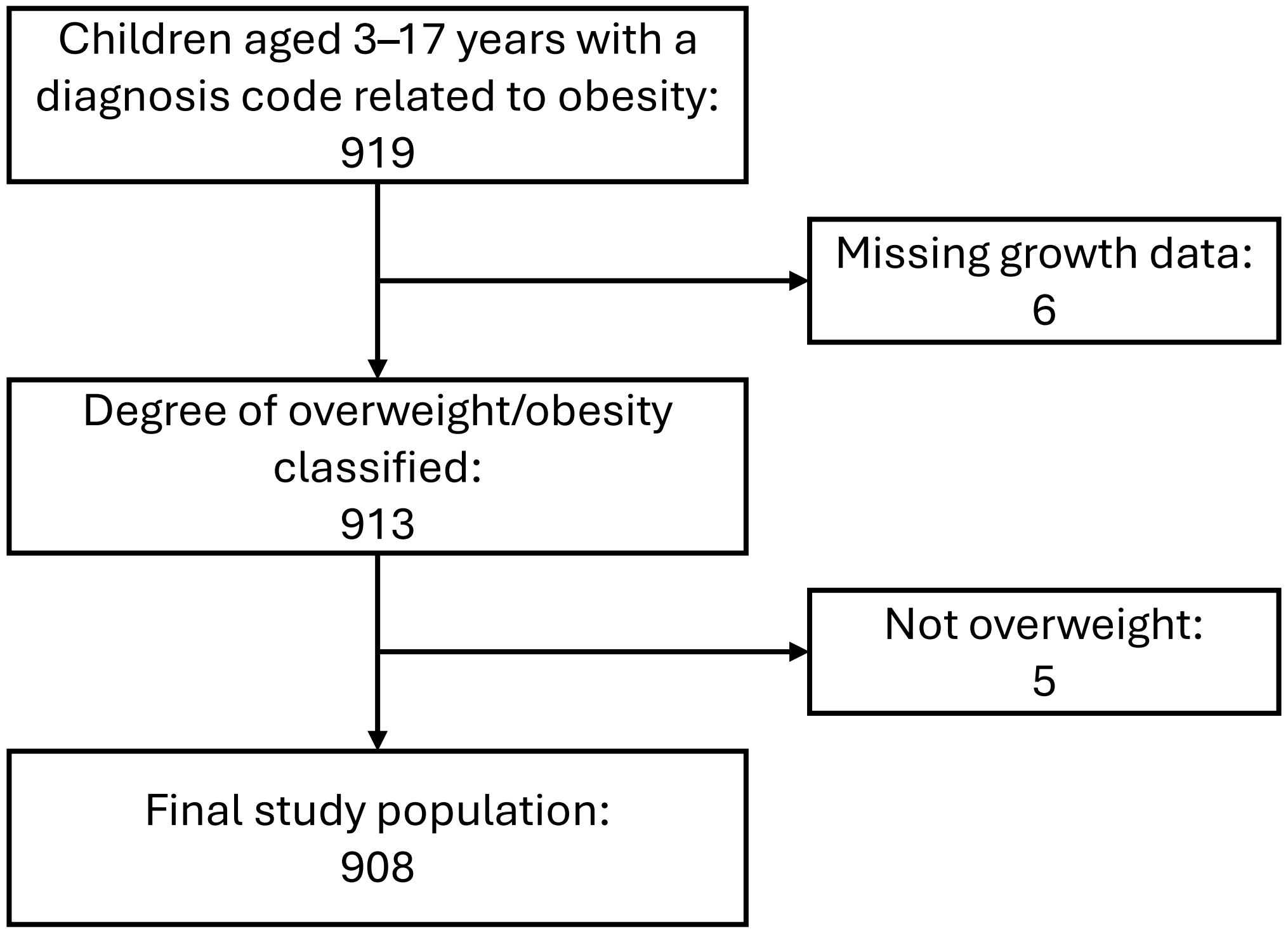
Children aged 3–17 years with a diagnosis code related to obesity: 919
Missing growth data: 6
Degree of overweight/obesity classified: 913
Not overweight: 5
Final study population: 908
